# Indoor Air Quality: An Overdue Awakening

**DOI:** 10.1021/acs.est.6c02998

**Published:** 2026-04-24

**Authors:** Linsey C. Marr, Richard L. Corsi

**Affiliations:** † Department of Civil and Environmental Engineering, Virginia Tech, Blacksburg, Virginia 24061, United States; ‡ Department of Civil and Environmental Engineering, 8789 University of California, Davis, California 95616, United States

**Keywords:** outdoor air quality, pollution, ultrapure air

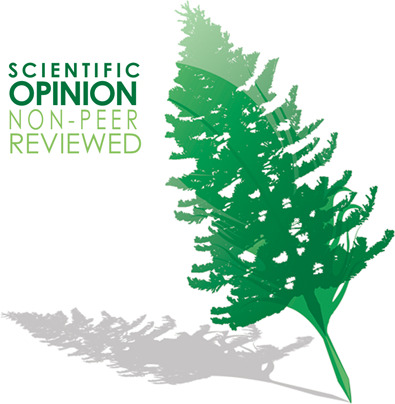

## Graphical abstract

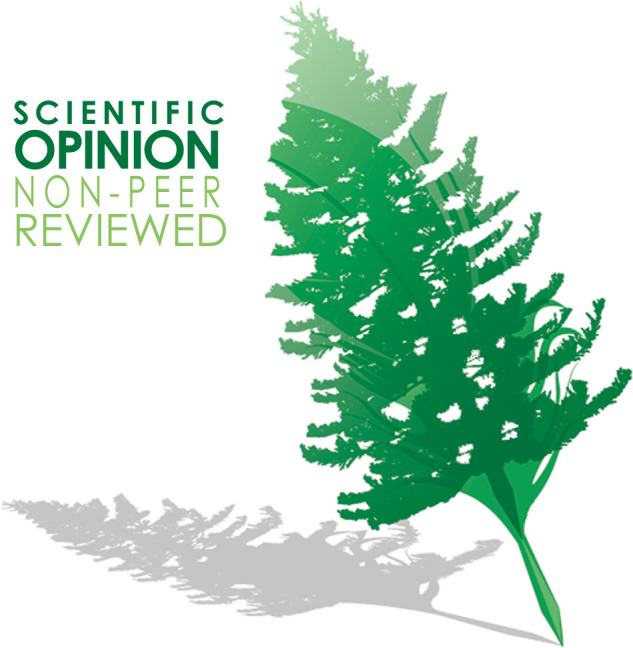

As *Environmental Science & Technology* celebrates its 60th anniversary, the U.K. Clean Air Act and U.S. Clean Air Act have reached similar milestones: 70 and 63 years, respectively. These laws have produced dramatic improvements in outdoor air quality and substantial benefits for human health. The benefits of the U.S. Clean Air Act are estimated to exceed costs by a ratio of more than 30:1.

Over this same period, knowledge about indoor air quality has improved tremendously, motivated in part by recognition that people in developed countries spend roughly 70 years of their lifetimes indoors, on average. Nevertheless, legislation, policies, and guidelines have been limited to radon and a few other special cases. Importantly, because outdoor and indoor atmospheres form a continuum, improvements in outdoor air quality have reduced indoor air pollution of outdoor origin. However, except for a few important pollutants, such as environmental tobacco smoke, improvements in indoor quality associated with pollutants generated indoors have been slow or nonexistent.

In 2000, the magazine *Environmental Manager* featured an article titled “Indoor Air Quality: A Time for Recognition”,^1^ which traced the evolution of modern indoor air quality problems and called for a Clean Indoor Air Act to address the substantial impact of poor indoor air quality on acute and chronic diseases. In the quarter century since that article was published, the recognition of airborne transmission of SARS-CoV-2 and increasing frequency of wildfires have added new dimensions to concerns about indoor air quality. A policy forum article in *Science* ^2^ and a Model Indoor Air Quality Act have presented a road map for progress on improving indoor air quality. Here, we tackle three issues that merit further consideration as researchers, policy makers, and the public navigate the route toward cleaner, healthier indoor air.

## Public versus Private Spaces

Whereas outdoor air is a public good, indoor air exists in both public and private structures and varies widely in quality from building to building, raising the question of how best to ensure clean air for the most people. We can look to the regulation of drinking water, food safety, and building fire safety as models, as these apply to entities that span the public–private spectrum. For example, in the United States, tap water that is treated by a utility must meet stipulations of the Safe Drinking Water Act. In contrast, water from private wells is not subject to monitoring and regulation, although the U.S. Environmental Protection Agency and state public health agencies provide guidance about the safety of well water. Radon, which is the second leading cause of lung cancer in the United States, could serve as a model for action around indoor air quality in private structures. We should prioritize spaces in which many people spend long periods of time. Schools are an obvious place to start, and under-resourced communities, where benefits may be greatest, should be prioritized with respect to both research and interventions.

## Ultrapure versus “Clean Enough” Air

For chemical contaminants, health risks generally decrease with reduced exposure. However, ultrapure air that is free of biological material (i.e., bioaerosols) may not be best for our long-term health. Even if we could eliminate all bioaerosols from outdoor air that infiltrates into structures, we would not be able to prevent exposure from indoor sources. Studies have shown that some exposure to microbes may be beneficial for training the immune system, reducing the incidence of allergies and autoimmune diseases.^3^ On the other hand, bioaerosols can trigger asthma attacks, so controlling bioaerosols in indoor air requires a delicate balance. Bioaerosols include pathogenic viruses and bacteria that are responsible for billions of respiratory infections and millions of associated deaths per year. With drinking water, we seek to eliminate as many pathogens as possible through a stringent coliform standard. With air, we may want to allow for some amount of biological matter, although research is needed to determine optimal exposures. Tuning the indoor microbiome will depend on public acceptance, and achieving the right balance will be challenging because individual variability in immune response means that what is optimal for one person could be life-threatening for another.

## Simple versus Complex Solutions

For many researchers, the natural inclination is to seek an optimal solution by pushing technological boundaries. Indeed, efforts are underway to develop affordable, real-time sensors for chemical and biological contaminants, whose readouts can be used to assess risk and trigger automated engineering-based interventions that restore healthier conditions, for example, as part of ARPA-H Building Resilient Environments for Air and Total HEalth (BREATHE). While such smart building systems may eventually be implemented broadly, like fire suppression systems, simpler solutions are available now. We do not need to measure everything. In the case of emissions from cooking, an infectious person, or other sources in a building, opening windows, and turning on exhaust fans can markedly improve indoor air quality. Filter-based portable air cleaners, including the do-it-yourself filter box,^4^ can effectively remove pathogen-laden respiratory aerosol particles within a room. Filters are also effective against wildfire smoke, particularly when opening windows and doors is undesirable. Given the enormous range of settings and availability of resources, we need to offer a menu of solutions, not a “one-size-fits-all” panacea. These include lower-cost sensors coupled with building control systems that optimize for thermal comfort, indoor air quality, and energy use; active and passive air cleaning technologies; and low-emitting materials, products, and practices that can be applied in new construction and under-resourced buildings.^5^

With these considerations in mind, we identify four major roadblocks to a Clean Indoor Air Act and suggest approaches to overcome them. First, in the United States and other countries, regulatory authority is unclear, often reflected in disaggregated and seemingly noncollaborative research and guidance between various government agencies. A lead authority on indoor air quality is needed to oversee greater collaboration and advancement. Second, building owners, managers, and occupants often have different priorities. A well-defined Clean Indoor Air Act and clear government authority could help align these priorities. Third, complexity in the variety of building ages, types, technologies, and occupant activities requires a flexible, heterogeneous approach to improving indoor air quality. Fourth, establishing what constitutes “clean enough” air will require consensus based on input from diverse disciplines, supported by further research involving, at a minimum, exposure, health, microbiological, physical, aerosol, and building scientists.

One hundred years ago, clean water was not a given. In 1900, the incidence of typhoid fever, a waterborne disease caused by *Salmonella* bacteria, was approximately 100 cases per 100 000 people in the United States. In 2006, the rate was only 0.1 case per 100 000 people, and the majority of cases occurred in international travelers. The combination of increasing awareness, new regulations, and advances in treatment technologies led to the expectation of clean water and the near elimination of waterborne diseases in some regions. One hundred years into the future, we hope that a similar revolution will have occurred for indoor air.
